# Implementation and evaluation of a model-based risk management process and service enhancement for home-based community care services amidst COVID-19 pandemic in Hong Kong: A mixed-method approach

**DOI:** 10.3389/fpubh.2023.1070182

**Published:** 2023-02-16

**Authors:** Caroline W. L. Yang, Alice N. T. Wan, Mable C. W. Kwok, Tai-Hing Lam, Agnes Y. K. Lai

**Affiliations:** ^1^School of Nursing, The University of Hong Kong, Hong Kong, Hong Kong SAR, China; ^2^Aberdeen Kai-fong Welfare Association Social Service, Hong Kong, Hong Kong SAR, China; ^3^School of Public Health, The University of Hong Kong, Hong Kong, Hong Kong SAR, China

**Keywords:** risk, management, COVID-19, service enhancement, non-government organization, social service, home-based, community services

## Abstract

**Background:**

The COVID-19 pandemic has greatly challenged all public social services, particularly home-based community care services (HBCCS). Aberdeen Kai-fong Association (AKA) is a non-government organization (NGO) in Hong Kong that systematically manages the challenges to HBCCS. This paper presents a practical example of the implementation and evaluation of the risk management process for HBCCS.

**Methods:**

Mixed-method design was used to evaluate the implementation of the risk management process in encountering the challenges from existing and potential problems to maintain and enhance HBCCS in four major areas amidst the pandemic. A cross-sectional questionnaire survey and three qualitative focus group interviews were conducted by AKA from 30 December 2021 to 12 March 2022 to collect staff feedback on the institutional risk management process in four areas.

**Results:**

109 HBCCS staff members (69% aged 40 years or above; 80% female) completed the questionnaire survey. For resource arrangement and staff training, over 90% of the participants agreed (including strongly agreed) that they had sufficient and reliable personal protective equipment and clear infection control guideline and effective training. Over 80% agreed they had safe working space and effective manpower allocation. However, only 75% agreed they had received emotional support from the organization. Over 90% agreed that the basic services were maintained for service continuation and enhancement, the service users and their families trusted the organization, and the provided services were adjusted according to users' needs. 88% agreed that the organization had obtained support from the neighborhood. For communication among stakeholders, over 80% agreed they had open discussions with the senior management team, and the senior management team was willing to listen. Twenty-six staff members joined the three focus group interviews. The qualitative findings corroborated the quantitative results. Staff appreciated the organisation's work to enhance staff safety and continue advancing services during this difficult period. Regular in-service training, updated information and guidelines to staff, and proactive phone calls to service users, especially the elderly, were suggested to enhance the quality of services.

**Conclusions:**

The paper could help NGOs and others encountering management challenges in community social services in diverse settings amidst the pandemic and beyond.

## Introduction

The COVID-19 pandemic has brought unprecedented and great challenges to all public social services. A good risk management process is vital for organisations' and sectors' service maintenance, enhancement, and assurance. Home-based community care has played a crucial role throughout the pandemic, meeting the most urgent social needs of vulnerable groups, such as the elderly and disabled. Effective and efficient risk management strategies can help organizations identify problems, analyse the needs and risks early, prioritize services, and act appropriately and promptly.

Global life expectancy has increased with modern medicine and technological advancements (1, 2). The aging population has led to an unprecedented rise in demand for healthcare and social services. To meet the caring needs of older people with multiple health problems and complex conditions, the Hong Kong SAR Government provides a range of home-based community care services (HBCCS), such as personal care, nursing care, rehabilitation services, meal and household cleaning services, carer support and emergency assistance through non-governmental organizations (NGOs) (3). These services aim to facilitate service users to continue living in the community for as long as possible, maintain their optimal level of function, improve their quality of life and ease the burden of services (3).

The Aberdeen Kai-fong Welfare Association Social Service (AKA) is one of the 61 non-government organizations (NGOs) running HBCCS. AKA provides a wide range of services, including home care, nursing and rehabilitation services for elders, people with disabilities and families in need in the Southern District of Hong Kong (4). Their goal is to facilitate this group of people and their carers to keep living in their community with dignity while receiving appropriate care. Furthermore, it incorporates a partnership with the neighborhood and local organizations to supplement the services and benefit the service users.

Hong Kong has experienced five waves of COVID-19 since December 2019 (5). By adopting the zero-COVID policy, the pandemic had successfully controlled with decisive border control, strict quarantine, and social isolation measures in the first four waves throughout the 2 years of 2020–2021 (6–9). Hong Kong reported fewer than 13,000 confirmed cases and 211 cases of death (10). However, the highly transmissible Omicron variant started the fifth wave in January 2022, and the number of confirmed cases escalated drastically (10). Over 42,000 confirmed cases were reported daily, with over 7,000 cases of COVID-19-related deaths during the peak of the fifth wave in March 2022 (11).

During the pandemic, the first and fifth waves are the most difficult periods for local citizens and social service providers because of resource constraints, social distancing measures and the reduction of public services to limited essential services and immediate suspension of face-to-face services. These brought enormous demands and burdens on health and social services and traumatic economic impacts (12, 13). The deteriorating social situations, such as expensive personal protective equipment (PPE), insufficient COVID-19 test kits, panic-buying of daily necessities, long queues for compulsory COVID-19 testing etc., have brought a tremendous threat to the daily living of residents (14, 15). As a result, the demands for HBCCS services dramatically increased, and organizations needed to adapt to the frequent infection control and related policy changes and adjust their services to meet the needs of their service users. Another major issue was the shortage of manpower in HBCCS providers. To control the spread of the virus, close contacts of COVID-19 patients were quarantined in quarantine centers or required to home quarantine for seven to 14 days, which varied during different phases of the pandemic (10). Therefore, many organizations did not have sufficient manpower to maintain their essential services when staff members were infected or quarantined.

**Figure 1:**
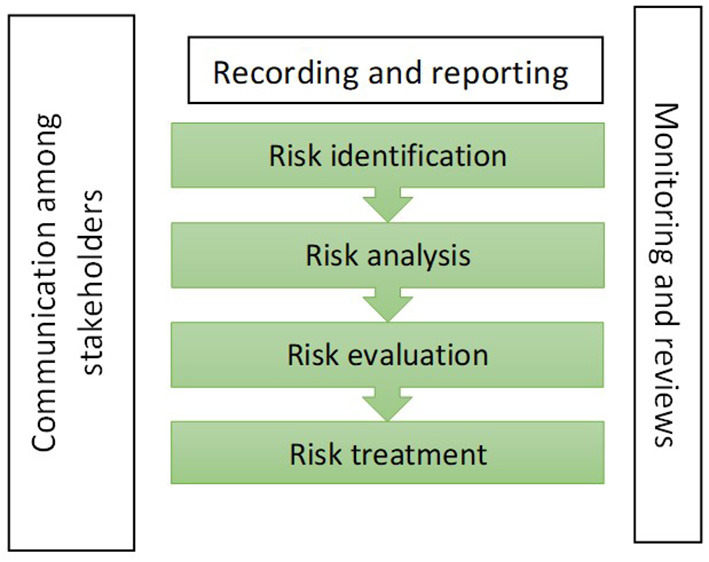
The elements of the risk management process.

The International Organization for Standardization (ISO) is a worldwide federation of national standards bodies which promotes using the risk management process to identify, analyse, and evaluate existing and potential problems and risks, and to effectively manage challenges and provide proactive measures in maintaining the services (16). Figure 1 shows the elements of the risk management process (16). Risk is defined as the effect of uncertainty on objectives (16). The risk management process is “a process that involves the systematic application of policies, procedures and practices to activities of communicating and consulting, establishing the context and assessing, treating, monitoring, reviewing, recording and reporting risk” (16, 17). The process includes risk identification, analysis, and evaluation to assess all sources of risk and risk treatment to handle and control the risks (16, 17). The processes are supported by effective communication and consultation, detailed recording and reporting and continuously monitoring and reviews (16, 17). Communication among stakeholders and professional consultations help stakeholders understand the identified risks and make decisions with relevant evidence. Through systematic risk management practices, a risk management culture can be developed within the organization in monitoring and managing risk, and the governance and performance of the organization can be improved (16, 18, 19).

We searched “PubMed” and “Social Service Abstract Search” for those articles published from 2020 to 2022 on 6 Jan 2023. Four groups of keywords were selected for searching relevant studies. The first set was a specific phenomenon “COVID-19.” The second set was the targeted population “Home-based care,” the third set was intervention “risk management” and the fourth set was the outcome “staff's experience/satisfaction.” It yielded 91 citations from the databases; none were duplicated records. Of the 91 studies, 18 were removed because they were literature reviews, conference proceedings, commentary, instructional materials, or guidelines, 6o were removed after the title and abstract screening, and one was removed after reviewing the content. Twelve studies reported the risk management support to home-based community care staff during the COVID-19 pandemic, including six studies with qualitative feedback (20–25), four studies with quantitative feedback (26–29) and two studies with mixed method (30, 31). Of these, three studies reported limited access to personal protective equipment (PPE) for home care staff and their service users in the early phase of COVID-19 and unclear guidelines on infection control (26, 27, 29). Halcomb et al. (27) addressed the insecure working atmosphere of primary healthcare settings in Australia at the start of the pandemic. Lethin et al. (26) reported how community organization support affected the mental health of home care staff in four European countries. Many healthcare activities were stopped because of social isolation measures (20, 23, 25, 28). Five studies (22–25, 29) reported telemedicine as one of the strategies for maintaining the home-based primary care service and its challenges, such as technical difficulties in approaching patients amidst the pandemic. Researchers also stated how community health workers adjusted the services to meet their needs, such as arranging case management outreach, coordinating elderly services and providing assistance to secure the basic needs of their home-based community care services (20, 21, 28, 30, 31). Mulligan et al. (31) evaluated the client satisfaction with mental health interventions, such as stress management, sleep hygiene etc. de Vries et al. (30) reported the mental distress of vulnerable groups and health professionals, and physical distancing affected the quality of life, mobility, and safety amidst the pandemic.

This paper aims to offer a practical example of multi-sector collaboration led by AKA and the mixed-method evaluation of the risk management process. Our research question was, “Did AKA HBCCS eligible staff agree that the risk management and service enhancement measures were effective?”. Besides, this paper demonstrates a step-by-step risk management process to manage COVID-19-related challenges and provides actionable suggestions for staff-centered training and service enhancement. The results might also help improve the effectiveness of the risk management process.

## Implementation of the risk management process

AKA used a modified ISO theoretical framework to implement a risk management process, including risk identification, analysis, and evaluation (32) to systematically encounter the challenges and potential problems at three (personal, interpersonal and institutional) levels in four major areas amidst the COVID-19 pandemic. The four major areas were: “Staff training and support” at the personal level, “Communication among stakeholders” at the interpersonal level, and “Space and resource” and “service continuation and enhancement” at the institutional level.

Table 1 shows the risk management process in four major areas of HBCCS in AKA during the pandemic. The four areas include (i) space and resources to prevent the spread of COVID-19; (ii) staff in-service training and support to promote professional knowledge and psychological support to enhance the institutional working atmosphere and staff morale; (iii) service continuation and enhancement, to continuously provide services to meet physical and psychosocial needs of their service users; and (iv) communication among stakeholders, to meet all relevant stakeholders regularly to listen to their voices, seek expert advice, connect with regional neighborhood organizations, institutions and local leaders and ask for collaboration, if necessary.

**Table 1:** The risk management process in four areas of home-based community care services in AKA amidst the COVID-19 pandemic.

**Area**	**Risk identification, analysis and evaluation**	**Risk treatment**
1. Space and resource	• Inadequate space to provide infection control training for staff and volunteers	• Liaised with nearby schools to use their classrooms for infection control training
	• Insufficient working space for staff which increased the risk of clustering	• Arranged clerical support staff to work from home • Home-based frontline staff were divided into sub-groups and relocated to different offices.
	• Shortage of manpower	• Allocated some staff members from other departments to those with insufficient workforce.
2. Staff in-service training and support	• Frontline staff were required to have home visits; they had a higher risk of exposure to COVID-19	• Regular infection control training and individual consultation • Provided showering facilities for staff after home visits • Sought expert advice from the infection control team of clinical partners • Delivered updated infection control guidelines to staff
	• Increased stress from frontline staff	• Purchased and delivered healthy foods and personal care products for free, e.g., air purifiers, to frontline supporting workers
3. Service continuation and enhancement	• Some service users and disadvantaged families did not have a quick and adequate supply of PPE	• Arranged volunteers to pack and deliver the personal protective equipment and delivered it to their service users for free, resident organizations and churches to support the community
	• Some service users lacked access to daily necessities because of the panic buying and hoarding of the public	• Served as a purchasing agent and delivered the daily necessities by staff and trained volunteers • Delivered the care packs to vulnerable people in the community by connecting with local leaders
	• Some service users could not maintain household hygiene	• Collaborated with AKA's corporate partners to provide free disinfection cleansing service to their service users
	• Some service users were unable to queue for long hours for the COVID screening	• Arranged special escort service for vulnerable groups • Provided delivery service to send specimens for COVID screening
	• Social distancing policy affected the mental health of the elderly	• Arranged volunteers to make regular phone calls to service users and vulnerable groups • Taught older adult service users to use online social media
4. Communication among stakeholders	• Uncertain of the effectiveness of the risk management measures	• Consulted professional parties and mid-level managerial staff • Arranged regular department meetings to meet with different levels of staff to collect their feedback • Connected with district neighbor associations and institutions, including nearby schools, and local leaders, for further collaboration

### Space and resource arrangement

To prevent the spread of COVID-19, the Government has imposed a series of preventive measures, including social distancing, suspension of non-essential services and closure of many public facilities, a vaccine pass scheme and compulsory testing at different periods during the pandemic. Despite the suspension of the non-essential services, the core services of HBCCS, like basic personal and nursing care, meal delivery and escorting services, must be maintained. Since the community hall of AKA had to be closed, AKA needed more space to provide infection control training to staff and volunteers. In this regard, AKA liaised with neighbor institutions, like the nearby schools to lend their classrooms (when school teaching was suspended) for infection control training. Moreover, to minimize the risk of virus exposure, AKA assessed the work arrangements among staff. The clerical support staff were arranged to work from home, and staff of home-based services were divided into sub-groups and located in different offices to reduce clustering.

Another major issue was a manpower shortage. Because of the rapid escalation of the confirmed cases, one-third of the HBCCS staff members could not work because of getting infected and mandatory quarantine. As the day-care center service was forced to be suspended, AKA allocated staff to those departments with insufficient manpower. Through interdepartmental collaboration, the organization had overcome much of the threat of manpower shortage.

### Staff in-service training and support

To continue the HBCCS, AKA recognized that the frontline staff might have a higher risk of exposure to COVID-19, especially for those who did home visits. To enhance the awareness and knowledge of COVID-19 and self-efficacy of self-protection, the organization conducted infection control training regularly and provided individual consultations to staff and volunteers as needed. The training and support helped equip staff and volunteers to deal with uncertain situations during service delivery. Moreover, AKA provided showering facilities after home visits to protect their staff. AKA sought expert advice from the infection control team of their clinical partners, monitored the local situations closely and delivered the latest infection control guidelines to protect the health of the staff. Despite providing PPE and infection control training to staff to minimize the risk of infection, some staff still had varying degrees of worriedness and fear of being infected. To strengthen staff morale and show care and concern to frontline supporting staff, AKA purchased and delivered healthy foods and personal care products, for example, air purifiers, to them as compliments, which positively reinforced the organization and staff commitment to persevere in serving the public.

### Service continuation and enhancement

Due to the massive local and global shortage of PPE, such as surgical masks and COVID rapid test kits (33, 34), AKA purchased PPE swiftly and delivered them to their service users for free. Besides, AKA noted that some low-income and disadvantaged families might also lack surgical masks and hygiene products. AKA arranged volunteers to pack the PPE and deliver them to the local community, including disadvantaged families, schools, churches, etc., to support the community in fighting the pandemic.

Apart from PPE, in the early stage of the pandemic, the Hong Kong public were panic buying and hoarding daily essentials and hygiene products, such as toilet paper, rice and bleach (15, 35). Such panic was more serious in the aged and disabled. Thus, AKA provided a special service to help with shopping and daily necessities. Furthermore, considering the lack of support for the vulnerable people, AKA also delivered the care packs, including bleach, detergent, liquid soap etc., to them *via* the local leaders. The local leaders were familiar with the deprived groups, particularly those living in the squatter areas in the districts, and they were most helpful.

Given the restrictions of social distancing, AKA recognized that some service users might be unable to maintain household hygiene. AKA collaborated with its corporate partners to provide free disinfection cleansing services to their service users, particularly the disadvantaged elders and families in the community.

Because of the compulsory test regulation, the Government requires any person present at designated places during the specified period to undergo a COVID-19 nucleic acid test. Some of the elderly were living alone or were disabled, so they could not queue for long hours for the mandatory COVID-19 screening. AKA arranged a special escort service for these service users to comply with the law. AKA also arranged delivery services, such as specimen collection packs and specimens after collection, to service users who needed to collect and send deep-throat salivary specimens for compulsory tests.

Due to the social distancing policy, many older people's mental and physical health were adversely affected. Therefore, AKA arranged two teams of volunteers to show their care and concern for their service users, vulnerable people, and their families. First, AKA arranged a team of trained volunteers to make regular phone calls to the service users and vulnerable groups to show their concerns and help identify any issues or problems that they needed help. They also made referrals to other organizations and followed up when necessary. Second, AKA arranged a team of youth volunteers to teach the older adult service users to use online social media to help them stay connected with relatives and friends and community service organizations, including AKA.

### Communication among stakeholders

Effective communication and consultation are crucial to improving the staff's understanding of risks and management processes. Regular department meetings were held, with formal and informal contacts with different levels of AKA staff to collect their feedback and opinions and establish organization rapport. AKA also established connections with district neighbor associations, institutions, and local leaders, facilitating collaboration and mutual help.

## Methods

### Study design

We used a mixed-method approach to evaluate the effectiveness of the risk management process and service enhancement measures in four major areas by conducting (i) a cross-sectional self-administered questionnaire survey and (ii) three focus group interviews.

### Recruitment procedures

A self-administered questionnaire was distributed to staff from 30 December 2021 to 28 February 2022 to collect feedback on the risk management measures. Participation was voluntary.

Invitations were sent to all HBCCS staff for the focus group participants, and they could join the focus group interviews voluntarily. The focus group interviews explored social workers' individual lived experiences in a group context, which might provide a greater understanding of the phenomenon under study. The Zoom interview link was sent *via* email or WhatsApp to those who agreed to join the online focus group interviews. Three 1.5-hour online focus group interviews were conducted on 23 Feb 2022 (2 groups) and 10 Mar 2022 (1 group). The interviews were moderated by the first author (CY), a university academic in nursing with a Master's degree in Nursing and over 20 years of clinical nursing and teaching experience. CY was responsible for asking questions using a semi-structured interview guide (Table 2). The last author (AL), another university academic in nursing and behavioral scientist with two doctoral degrees in Nursing and Public Health and over 20 years of clinical nursing and teaching experience, was responsible for monitoring participants' responses and ensuring active participation. A research assistant with a master's degree in applied psychology was responsible for taking notes during the interviews to record important points. The focus group interviews were tape-recorded and transcribed verbatim. Questions were structured chronologically to aid recall and were phrased to provide scope for additional areas to emerge. The questions focused on staff's experiences with COVID-19, particularly the challenges at work and support needs amidst the pandemic in Hong Kong in the past 2 years.

**Table 2:** The semi-structured interview guide for focus group interviews.

1.	How the COVID-19 pandemic affects your work? Especially when the protective resources were severely insufficient, what was your feeling during that moment?
2.	Any special needs from the service users? How do you respond to their needs?
3.	What are the challenges at work? How do you solve these challenges?
4.	In your experience, have you ever cared for service users who suffered from COVID-19? If yes, can you please share your experience?
5.	How do you comment on the risk management measures of the organization during the pandemic?
6.	How was the service affected during the pandemic?
7.	Did you ever feel stressed during the pandemic? Why?
8.	Please tell me what support you need at work.

### Participants

All HBCCS staff from the AKA Social Service were invited to join the study. The inclusion criteria were: (i) aged 18 years or older, (ii) able to read Chinese and speak Cantonese. The exclusion criteria were: (i) non-HBCCS staff of AKA and (ii) those who cannot read Chinese and speak Cantonese. The ethical approval of this study was approved by the Institutional Review Board (IRB) of the University of Hong Kong/Hospital Authority Hong Kong West Cluster (HKW IRB reference number: UW21-781). Written informed consent was obtained.

### Measures

#### The survey

The research team designed an outcome-based questionnaire in Chinese, including two experienced social workers (AW and MC) and two academics with extensive experience in conducting service and training evaluations. We invited two AKA HBCCS frontline staff to answer and comment on the draft and then modified the questions according to their feedback to ensure the adequacy and understandability of the questionnaire.

Participants were asked to indicate the extent of agreement with statements in the four areas of risk management by using a six-point Likert scale, ranging from “1 = strongly disagree” to “6 = strongly agree.” A higher score indicates greater satisfaction. The four areas of risk management measures were:

***(i) Space and resource arrangement:*** Three statements were on the supply of PPE, the arrangement of a safe working venue, and the manpower allocation (for example, “The organization provided reliable and effective personal protective equipment).”

(ii) ***Staff in-service training and support***: Three statements were on the infection control training, guidelines and institutional support to staff (for example, “The organization provided emotional support and encouragement to staff.”).

(iii) ***Service continuation and enhancement***: Five statements were on the sustainability and enhancement of services, including service maintenance, trust from service users and their families, adjustment to user-orientated service, encounters of service users' needs and support from the neighborhood (for example, “The organization adjusted the home care services based on the needs of the service users.”).

(iv) ***Communication among stakeholders:*** Two statements were on the communication within the organization throughout the risk management process (for example, “The senior management team is willing to hear the feedback from staff.”).

The survey also included five items on demographic information: sex, age, education level, years of service in social services and their role in the organization.

#### The focus group interviews

Two researchers (CY and AL) trained in qualitative methods conducted focus group interviews on 7–10 participants using a semi-structured interview guide and Zoom video conference software. The interview questions focused on (i) how the pandemic affected their work, (ii) the risks that they experienced at work, (iii) comments on the risk management measures of the organization, and (iv) what their support needs are.

### Statistical analysis

#### Data analysis

All statistical analyses were performed with SPSS for Windows (version 28). Participants with missing data were excluded. Data were presented in frequencies and percentages, and continuous data were presented as mean and standard deviation. Chi-squares test was used to assess any difference in staff characteristics between those who joined and those who did not join the focus group interviews.

For the focus group interviews, all contents were audio-taped and transcribed verbatim in Cantonese to capture every nuance of expressions unique to the dialect. At least (10%) of the transcripts were checked against the recordings. Two project team members who had attended all the interviews processed coding. Transcripts were analyzed by thematic content analysis, following the guidelines recommended by Morse and Field (36). Each transcript was analyzed sentence by sentence and coded for the participants' meanings.

#### Techniques to enhance trustworthiness

Different strategies were used to enhance the trustworthiness of the findings, including credibility (the truthfulness of data), dependability (the stability of data), confirmability (the congruence of data) and transferability (the applicability of data). To enhance study credibility, member checking was conducted by asking participants (one respondent from each focus group interview) to review the transcripts from interviews they participated in and give feedback on emerging interpretations to ensure a good representation of their realities. Two researchers analyzed each interview. Peer debriefing was then held to review the consistency of identified information with other co-investigators. To enhance study dependability, the description of the coding and the descriptions of themes were checked and reconfirmed by a research staff member who was not involved in data collection. To promote study confirmability, an audit trail was conducted by making field notes when conducting interviews to allow tracing of the course of work. Moreover, we reported the study design details, investigators' characteristics, participants' characteristics, sampling strategies, data collection and analysis procedures to promote study transferability.

Mixed-method triangulation design was used to interrelate and interpret the qualitative and quantitative data to validate the results (37).

## Results

All 119 AKA HBCCS eligible staff were invited to join the study. Three refused to join. One hundred and sixteen staff signed the consent form and completed the questionnaire. Eight did not provide complete data and were excluded. Those who agreed to participate in the survey were invited to join the focus group interviews. Finally, 26 joined the focus group interviews.

### Participants

Table 3 shows that 80% were female, 69% were aged 40 years or above, and 69% had secondary or below education. 76% were supporting staff (care workers or health assistants), and 25% were professional staff (social workers, nurses, physiotherapists, and occupational therapists). 55% had worked in community care settings for 5 years or more. Of the 26 (77% female and 73% aged 40 years or above) who joined the focus group interview, 69% were supporting staff, and 58% had working experience in HBCCS for 5 years or more. We found no significant differences in demographic characteristics between those who joined and did not join the focus group interviews.

**Table 3:** Characteristics of survey participants.

	**All** **(*n* = 109)**	**Joined focus group** **(*n* = 26)**	**Did not join a focus group** **(*n* = 83)**	***P*-value**
	***n* (%)**	***n* (%)**	***n* (%)**	
Sex				0.67
Female	87 (79.8)	20 (76.9)	67 (80.7)	
Male	22 (20.2)	6 (23.1)	16 (19.3)	
Age group				0.29
18–40 years	34 (31.2)	7 (26.9)	27 (32.5)	
40 years or above	75 (68.8)	19 (73.1)	56 (67.5)	
Educational level				0.66
Secondary school or below	75 (68.8)	15 (57.6)	60 (72.3)	
University level or above	34 (31.2)	11 (42.3)	23 (27.7)	
Roles in the organization				0.41
Supporting staff^a^	80 (75.5)	18 (69.2)	62 (74.6)	
Professional staff^b^	26 (24.5)	8 (30.8)	18 (21.6)	
Years of service in community care				0.07
Below 5 years	49 (45.0)	11 (42.3)	38 (45.7)	
Five years or above	60 (55.0)	15 (57.7)	45 (54.3)	

^a^Supporting staff included care workers and health assistants.

^b^Professional staff included social workers, nurses, physiotherapists, and occupational therapists.

Chi-square test by P-value for the difference between 2 groups.

### The findings

**Figure 2:**
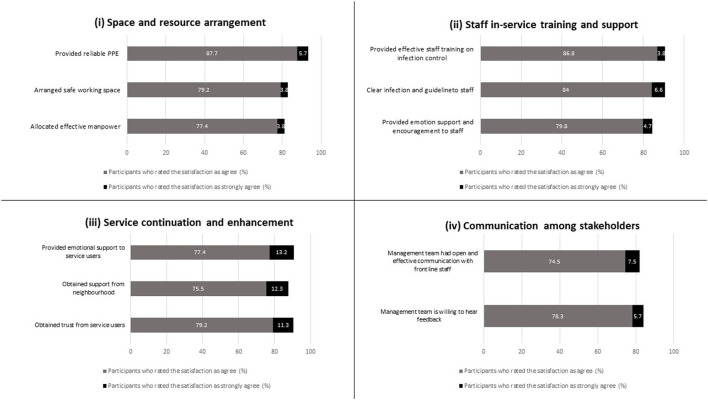
Feedback from survey participants on four areas of institutional risk management amidst the COVID-19 pandemic.

Figure 2 shows the feedback on four areas of the risk management process and measures amidst the COVID-19 pandemic, which included (i) space and resource arrangement, (ii) staff in-service training and support, (iii) service continuation and enhancement, and (iv) communication among stakeholders. Table 4 shows each area's mean score and standard deviation (mean ± SD).

**Table 4:** Mean scores on the extent of agreement on risk management measures amidst the COVID-19 pandemic (*n* = 109).

		**All participants**
		**(*****n*** = **109)**
		**Mean** ±**SD**
**Space and resource arrangement**		
1.	The organization provided reliable and effective PPE.	4.99 ± 0.35
2.	The organization arranged a safe working space for staff to work.	4.83 ± 0.55
3.	The organization allocated the workforce effectively to maintain the service.	4.83 ± 0.48
**Staff in-service training and support**		
1.	The organization provided effective infection control training to staff.	4.94 ± 0.36
2.	The organization had clear infection control guidelines for staff.	4.97 ± 0.39
3.	The organization provided emotional support and encouragement to staff.	4.79 ± 0.53
**Service continuation and enhancement**		
1.	The organization maintained basic services.	5.07 ± 0.54
2.	The organization adjusted their service based on the needs of the service users.	5.00 ± 0.53
3.	The organization provided service to fulfill the emotional needs of the service users.	5.03 ± 0.51
4.	The organization obtained support from the neighborhood.	4.98 ± 0.62
5.	The organization obtained trust from the service users and their families.	5.03 ± 0.49
**Communication among stakeholders**		
1.	The management team had openly and effectively communicated with frontline staff.	4.88 ± 0.52
2.	The management team is willing to hear feedback from staff.	4.88 ± 0.50

Higher scores indicate higher satisfaction: 6-point Likert scale: 1 = strongly disagree; 2 = disagree; 3 = slightly disagree; 4 = slightly agree; 5 = agree; 6 = strongly agree.

#### Space and resource arrangement

Ninety four percentage of participants strongly agreed or agreed that they had sufficient and reliable PPE from the organization (mean 4.99 ± 0.35). 84 and 81% strongly agreed or agreed that a safe working space was arranged for work (mean 4.83 ± 0.55) and manpower was allocated effectively to maintain the service (mean 4.83 ± 0.48), respectively.

In the focus group interviews, some participants expressed that the organization supplied PPE and COVID rapid test kits for them at work throughout the pandemic. Moreover, some participants wanted the organization to provide masks with higher levels of protection at work, especially during the fifth wave of the pandemic.

“*Actually, for supplies, the organisation provides us, like gloves, hand rubs, most of the supplies they give us are OK!” (Participant 12, a female care worker)*.

“*The organisation provides the rapid test kits for colleagues, and I think that this is a good thing” (Participant 21, a female social worker)*.

“*I think that right now what we need the most is high-quality masks (KF94) for us to use… Because the fifth pandemic wave is so severe and infectious, colleagues of other departments were also infected” (Participant 2, a female care worker)*.

On the other hand, owing to the global shortage of PPE during Wave 1, a nurse manager expressed understanding that the organization had put much effort into sourcing PPE for staff despite facing many difficulties in procurement of PPE and the high price of PPE.

“*It was hard to purchase goods (of PPE) two years ago…. Going to foreign websites to order (PPE) in the middle of the night…, one protective suit cost more than a hundred (Hong Kong; US$1*=*HK$7.8) dollars, and even though it was expensive, we still had to buy it to support and protect our frontline staff” (Participant 20, a female nurse manager)*.

Some participants also expressed the problems of manpower shortage during the peak of Wave 5. Still, the problem was solved by inter-departmental coordination and cooperation to minimize the work pressure and maximize the services in helping the elderly during such a critical moment.

“*We lost one-third of our manpower (during the peak surge of Omicron, Wave 5), but another team supported us” (Participant 23, a male social worker)*.

“*We allocated manpower from other departments (forced to suspend the service) to some departments with insufficient manpower... Although different departments provided different types of services since the pandemic was so severe, we helped and complemented each other internally in those departments that were severely affected… we integrated service management to maximise the facilitation… since we're helping the elderly now, who need our services a lot” (Participant 19, a female social worker)*.

#### Staff in-service training and support

Ninety one percentage of the participants strongly agreed or agreed that the infection control training (mean 4.95 ± 0.36) and guidelines offered by the organization were effective and clear (mean 4.97 ± 0.39), and 75% of the participants strongly agreed or agreed that they received emotional support and encouragement from the organization (mean 4.79 ± 0.53).

Some focus group participants agreed that the infection control training could strengthen their awareness of infection control and help protect themselves and others. However, a participant expressed her frustration with the great outbreak of Omicron, as she needed to update the infection control guidelines frequently and adequately and explain them to the staff. Still, the government's infection control and quarantine guidelines were unclear.

“*The in-house (infection control) training was useful. At least our awareness of keeping clean and awareness about the pandemic is strengthened every day… our department head also did online training for us (on top of the official training)” (Participant 1, a female care worker)*.

“*It (the infection control training) let us learn how to protect ourselves and the importance of protecting others” (Participant 6, a female care worker)*.

“*Because of the great outbreak of Omicron variant, no one knows who will be infected… and I couldn't reply to staff when I was asked to provide some information. I had to ask my friends (doctors in charge of the infection control team) who had up-to-date information, or I attended regular meetings in the hospital to get the newest information and explain it to my colleagues…… I'm not worried whether I will get the virus or not, but rather the risk of the team getting infected!” (Participant 20, a female nurse manager)*.

Moreover, some participants appreciated the organization for delivering healthy drinks, food, and care products to show concern.

“*This (delivering healthy food and drinks to frontline staff) is pretty good for morale, I think it can boost morale. At least someone cares about us, the frontline staff” (Participant 3, a female care worker)*.

“*Like today, the organisation gave us an air purifier, and we felt warm inside! I think it's a kind of encouragement” (Participant 2, a female care worker)*.

#### Service continuation and enhancement

Ninety one percentage of participants strongly agreed or agreed that the basic services were maintained (mean 5.07 ± 0.54), and the service users and their families trusted the organization (mean 5.03 ± 0.49). 88% and 90% strongly agreed or agreed that the organization obtained support from the neighborhood (mean 4.98 ± 0.62) and adjusted the service based on the needs of the service users (mean 5.0 ± 0.53), respectively. Furthermore, 90% strongly agreed or agreed that they fulfilled the emotional needs of their service users (mean 5.05 ± 0.51).

Some focus group participants expressed concerns about the influences of the frequently changed government policies on the elderly. These policies affected the daily lives of and placed much stress on the elderly. The staff tried their best to help the elderly cope with the challenges.

“*They (the elderly) were forced to go for COVID compulsory screening, but they couldn't get tested after lining up for 6-7 hours, so it's difficult for some of the elderly!” (Participant 22, a female social worker)*.

“*Two years ago, sometimes the elderly called the centre to ask, “What should I do? I can't buy any masks!”. Recently (during Wave 5), they could not buy any food because the shelves at the supermarkets were empty, so we arranged services to help the elderly to buy daily necessities” (Participant 22, a female social worker)*.

“*… for our group of social workers, we all needed to check and trace the latest information and policies of the government, and see which buildings have to undergo compulsory testing… we had to call and see when the elderly were preparing to queue, and we had to think of how to arrange their meals… and we must help them adapt to this environment” (Participant 21, a female social worker)*.

Some not only made many efforts to meet the physical needs of the elderly but also provided proactive phone call supports to comfort them and detect any potential problems.

“*I have noticed that during this pandemic, the elderly's emotions fluctuated a lot… like yesterday, we received a lot of phone calls from the elderly as they were scared by the alarm of unprecedented emergency alert via the mobile phones from the Government… I observed that no matter whether it is a piece of news or the ever-changing policies of the government, it would bring many worries to the service users. Hence, the role of a social worker is to comfort the elderly and the carers' emotions. We spent more time than usual on these” (P22, a female social worker)*.

Owing to government policies on pandemic control, the home-based rehabilitation service was forced to suspend. To prevent the worsening of the health condition of service users, the rehabilitation service was transformed from face-to-face to online exercise classes to continue the service with the help of the family members of the elderly.

“*Under the pandemic, many service users have stopped the rehabilitation services… we found out that their situation (cognitive and movement abilities) was worse than before. After the long waves of this pandemic, we tried to find ways to help them each time. Although the services were suspended, we would find some service users with online devices that could let them have a Zoom meeting at home, and we would exercise with them over Zoom. Maybe some families don't have these devices; we prepared several short videos and sent the link to them through WhatsApp or email to the elderly's families; they can do exercises together at home” (Participant 25, a female physiotherapist)*.

Other than how to enhance and extend the HBCCS, some participants expressed their dedication to serving the community.

“*We need to face the virus calmly; the community needs us; we definitely shouldn't be afraid!” (Participant 3, a male driver)*.

“*… at work, I am not very worried (about getting infected). I'll do my best and whatever I can” (Participant 8, a female care Worker)*.

“*… we have to be positive since we're helping the elderly now, who need our services a lot. Even though it may not be perfect, we'll do what we can!” (Participant 19, a female social worker)*.

#### Communication among stakeholders

Eighty two and eighty three percentage of participants strongly agreed or agreed that they had open discussion and communication with the senior management team (mean 4.88 ± 0.52), and the senior management team was willing to listen (mean 4.88 ± 0.5), respectively.

Some focus group participants highlighted the importance of communication among staff to build rapport and trust and the importance of being a role model and having a good team spirit.

“*I remember using much time to explain (infection control measures and guidelines) to the frontline staff… I did a lot to reassure my colleagues; I didn't only care about their work, I also cared about their health, and did what I could to be well-prepared for the challenges, which helped build trust” (Participant 20, a female nurse manager)*.

“*I'll have to be a role model and do my best so that they can follow us as an example. We're a team; this is teamwork” (Participant 23, a male social worker)*.

## Discussion

This is the first paper to report the implementation of “risk management process,” the systematic mixed-method evaluation of space and resources arrangement, staff in-service training and support, service continuation and enhancement, and communication among stakeholders. We have also made some actionable suggestions for in-service training and service enhancement from the staff's perspective.

Regarding space and resource arrangement, the shortage of PPE during this pandemic was a grave global issue. Our participants responsible for purchasing PPE also faced difficulties sourcing them because of the global shortage and the expensive prices. This is consistent with others' findings that the increased demand for facemasks by the public caused price acceleration and supply constraints to frontline healthcare professionals (38, 39). At the same time, some participants expected the organization to provide masks with the highest standard for them. This might be due to the inconsistent guidelines across regions and the frequently changed guidelines. Therefore, governments and public health agencies should give rational recommendations on the appropriate level of face mask use (39) and frequent updates and clarification (40).

To encounter the limited manpower and resources, AKA made good use of its neighborhood and social networks to collaborate with neighborhood institutions and district organizations, such as nearby schools, district leaders, medical partners, and volunteers to continue or adjust the existing services and provide emergency services amidst the pandemic. Good neighborhood and social networks help create useful connections, linkages and potentials within and among the community, organizations and society, facilitate resource mobilization (41, 42), and widen the support and encourage solidarity (42, 43). Researchers suggest that NGOs could maintain their activities by having peer support to overcome social risks and unexpected situations, such as disasters and health crises (44, 45). The mutual help of the local community networks can foster the quality of life and better living of the elderly (46), empower NGOs to build capacities to keep serving the community (43) and ensure the vulnerable and disadvantaged receive needed support and assistance (42).

Regarding staff in-service training and support, high satisfaction with the in-service infection control training was reported. The purpose of the in-service training was not only to focus on infection prevention but also to enhance the self-efficacy of staff to educate their service users and caregivers (47). Training is crucial for staff career development and job satisfaction (48). Besides, regular updates of infection control guidelines are also important during the pandemic (49). The uncertainty and unpredictability might affect one's intolerance of uncertainty (50) and cause fear and anxiety (51). Therefore, prompt and effective communication is one of the essential strategies for all government departments, institutions, and professional bodies in responding to the ongoing pandemic of COVID-19 (52). Emotional support and encouragement to staff, such as healthy drinks and care products, were given to staff by the organization. These actions can make staff feel valued, build loyalty and commitment, and minimize the risks of burnout (53, 54).

Regarding service continuation and enhancement, most participants agreed that the essential HBCCS service had been maintained, and the service was adjusted, extended, and enhanced to meet the needs of the service users. Proactive phone calls could provide psychological support and comfort to the elderly. Such service enhancement process involved teamwork, inter-departmental collaboration and rapport among staff which facilitated swift response during the critical time of COVID-19. This is consistent with other findings in the literature: interprofessional participation, trust and collaboration within teams can empower positive changes in services (55, 56). Furthermore, some focus group participants reported their dedication to serving the vulnerable, demonstrating significant commitment and goodwill to the community in response to the COVID-19 crisis (55).

In communication among stakeholders, most participants reported that the organization maintained effective communication. Communication is one of the core elements in developing a workplace culture of respect and trust (57). In addition, staff who found that their voices were recognized were more likely to have higher job satisfaction and feel empowered and recognized (58).

The study's strength was the use of both qualitative and quantitative data to enrich the understanding of the staff feedback on the management process amidst the pandemic. We suggest using a step-by-step risk management process (16) to manage those challenges from COVID-19 or others. Besides, our mixed method triangulation design can enhance the validity, reduce bias, and provide insights into the real situation of HBCCS during the COVID-19 pandemic (59, 60). Our questionnaire could be adopted or adapted for evaluating risk management processes in other community services or institutions.

However, our study had several limitations. First, we only showed the implementation of the risk management process in one NGO, which might limit the generalizability of the findings. Organizations might have different values, beliefs, human behaviors, cultures and dynamics; thus, the feasibility, applicability and effectiveness of using this model of risk management process might vary (61). Second, because validated questionnaires were unavailable, we developed our outcome-based questionnaire to assess staff feedback. We measured perceptions only, which might not reflect the actual situations. Individuals' perceptions can be influenced by their personality and self-perception (26, 62). Third, the subjects of this study were the organisation's staff, and some might not express their opinions freely. Social desirability bias might have exaggerated the positive findings. Therefore, an evaluation conducted by a third party may provide more reliable results.

## Conclusions

This paper offers a practical example of implementing and evaluating an NGO's step-by-step risk management process, providing continuous enhancement of home-based and community services during the pandemic.

Our paper demonstrates a step-by-step risk management process to systematically manage COVID-19-related challenges, evaluate staff feedback to understand staff and service needs better, and provide actionable suggestions for staff-centered training and service enhancement. This example might be helpful to others encountering management challenges in community social service challenges in diverse settings and services amidst the pandemic and beyond.

## Data availability statement

The raw data supporting the conclusions of this article will be made available by the authors, without undue reservation.

## Ethics statement

The study was approved by the Institutional Review Bard of the University of Hong Kong/Hospital Authority Hong Kong West Cluster (HKU/HA HKW IRB: UW 21-781). The patients/participants provided their written informed consent to participate in this study.

## Author contributions

CY contributed to the conception and design of the study, supervising data collection, data analysis, and manuscript drafting. AL contributed to the study's design, statistical analysis, and manuscript drafting. AW contributed to the conception and design of the study and recruitment strategy. MK contributed to the data collection strategies, commenting, and evaluating the manuscript. T-HL contributed to the conception and design of the study, drafting, and editing the manuscript. All authors read and approved the final manuscript.
